# The Contribution of FMRP to the Development of Speech and Vocabulary in Young Boys with Fragile X Syndrome: A Retrospective Examination

**DOI:** 10.3390/children12020245

**Published:** 2025-02-18

**Authors:** Stephen R. Hooper, John Sideris, Deborah R. Hatton, Joanne R. Roberts

**Affiliations:** 1 Department of Health Sciences, School of Medicine, University of North Carolina-Chapel Hill, Chapel Hill, NC 27519, USA; 2 Department of Occupational Therapy, University of Southern California, Los Angeles, CA 90007, USA; sideris@chan.usc.edu; 3 Frank Porter Graham Child Development Institute, University of North Carolina-Chapel Hill, Chapel Hill, NC 27519, USA

**Keywords:** FMRP, Fragile X Syndrome, articulation, speech production, language development, expressive vocabulary, receptive vocabulary

## Abstract

Background/Objectives: This study examined the development of speech, expressive vocabulary, and receptive vocabulary in boys with Fragile X Syndrome (FXS), with a focus on the contribution of the Fragile X Messenger Ribonucleoprotein (FMRP), while controlling for the effects of nonverbal IQ, maternal education, and Autism status on the development of these skills. Methods: Participants included 45 boys with full mutation FXS, ranging in age from 2.9 to 14.0 years, who were subdivided into those with FXS only (FXS-Only) and those with FXS and Autism (FXS-Autism). Speech, expressive vocabulary, and receptive vocabulary skills were assessed over three years for each participant. Results: There was a significant relationship between each of the outcome measures and the child’s nonverbal mental level, and between for both outcome measures of vocabulary and Autism status, but these relationships were moderated by the level of FMRP. Specifically, higher levels of FMRP seemed to increase the relationship between developmental level of speech, receptive, and expressive vocabulary for boys with FXS with and without Autism; however, at lower levels of FMRP, these relationships seemed to weaken significantly for both groups. Conclusions: These findings implicate increased complexity in the relationship between various contributors to the rates of growth of speech, expressive vocabulary, and receptive vocabulary in boys with FXS, with FMRP being a key variable potentially moderating the relationship between nonverbal abilities, Autism status, and speech and vocabulary development.

## 1. Introduction

Fragile X Syndrome (FXS) is the most common form of inherited intellectual disability that is created by a trinucleotide repeat expansion (cytosine-guanine-guanine–CGG) at Xq27.3 on the upper end of the Fragile X Messenger Ribonucleoprotein gene (FMR-1). In individuals with full mutations, the FMR-1 gene becomes methylated, and as a consequence the associated FMR-1 protein is not present or poorly/abnormally expressed. The presence of this critical RNA binding protein appears essential to normal brain functioning, while its absence contributes to aberrant underlying neurobiological functions such as regulation of messenger RNA, synaptic engagement, and regulation of numerous ionic channels that can be seen in many individuals with FXS [1,2,3].

The resulting deficiency of the FMR1 protein is responsible for the physical and neurodevelopmental characteristics of many individuals with FXS [4]. Some of these characteristics include the presence of intellectual disabilities and slowed developmental progress [5], behavioral and adaptive difficulties [6], and an array of neurocognitive problems (e.g., memory, executive functions) [7]. While the impact of differing levels of FMRP may not be as dose-dependent as previously thought [8], and its associations with IQ and other cognitive functions have been mixed [8,9,10], FMRP still remains a key factor in our understanding of the functioning of individuals with FXS. In that regard, speech and language functions have been of keen interest due to their importance to overall communication capabilities, and they have generated a significant amount of scientific inquiry in young males with FXS; however, how FMRP relates to the development of speech and language skills in young boys with FXS remained understudied.

### 1.1. Speech and Language Functions in Young Boys with FXS

Given the high prevalence of intellectual disabilities in young boys with FXS, it is not surprising that they also would be expected to manifest a variety of communication difficulties and that this area has demonstrated a steady stream of new information for the past two decades [11,12,13], with efforts spanning speech output, expressive language [14,15,16], receptive language [17], and phonological process [18]. In general, young boys with FXS have been described as showing moderate to severe language delays, with their receptive language abilities being generally better developed than their expressive language abilities [19,20].

Roberts et al. [20] reported that receptive language gains reflected about one-half the rate of typical development over time, while expressive language gains occurred at about one-third the rate. Roberts et al. [21] also examined whether boys with FXS with and without Autism, boys with Down syndrome, and younger mental-age matched typically developing boys differed in their patterns of speech production, receptive vocabulary, and expressive vocabulary. They reported that boys with FXS without Autism did not differ from the younger typically developing boys in their speech, expressive vocabulary, or receptive vocabulary, and that they also scored higher in receptive vocabulary than the boys with Down syndrome at similar nonverbal developmental levels. Further, Roberts et al. [21] noted that the boys with FXS and Autism scored lower in expressive vocabulary than the younger typically developing boys after adjusting for nonverbal cognitive abilities. This finding is important as FMRP has been linked to a number of genes associated with Autism, suggesting that FMRP may be of particular interest in individuals with both FXS and Autism [22,23]. While the level and pattern of these language abilities appears to differ across different developmental disabilities, such as Down Syndrome and Autism [21,23], specific molecular contributors to these longitudinal outcomes remain unclear at this time.

### 1.2. Speech and Language Functions and FMRP in Young Boys with FXS

Given its importance in brain development and brain functioning, the relative lack of FMRP appears to be a strong candidate for accounting for some of the deficits in speech, expressive vocabulary, and receptive vocabulary development in young boys with FXS which, in turn, can create downstream difficulties in functions such as social engagement, language pragmatics, and overall learning. While developmental studies of FMRP expression over time in children remain relatively non-existent, there are potential biological pathways suggesting a relationship between FMRP and behavior. Specifically, FMRP is known for its involvement in synaptic and dendritic plasticity and function, the myelination process and white matter development, and the structural organization and structure of the brain [24]; however, the association of these underlying neurodevelopmental processes related to FMRP and speech and language development, or phenotypic presentation more generally, remains unknown. Indeed, a few early studies have found variations in FMRP levels to explain some variability in language functioning in children with FXS [25,26,27]; however, more recent studies have reported FMRP levels to be not significantly related to language-based outcomes such as parent ratings of communication and measures of expressive and receptive vocabulary [28]. To our knowledge, there have been no studies that have addressed this relationship of FMRP with the development of basic communication skills over time in young boys with FXS, and this may serve as a first step to understanding biological pathways between FMRP and speech and language functions in boys with FXS.

### 1.3. Current Study

To address this gap in the literature, we accessed a retrospective longitudinal data set on young boys with FXS. These data would permit our preliminary examination of the specific contributions of FMRP to the rates of growth of speech, expressive vocabulary, and receptive vocabulary in a well characterized sample with the full mutation. Specifically, we asked whether FMRP levels would differentially affect the rates of growth of speech, expressive vocabulary, and receptive vocabulary over a three-year time period after controlling for key covariates (e.g., nonverbal IQ, maternal education, Autism status). It was hypothesized that FMRP would be positively related to the development of these speech, receptive vocabulary, and expressive vocabulary communication skills, with perhaps the interaction of low levels of FMRP with Autism status reflecting a differential pattern of influence (i.e., performing lower in core speech and language skills than those without Autism and a relatively higher level of FMRP).

## 2. Materials and Methods

### 2.1. Participants

All participants were part of a larger longitudinal study examining speech and language development in young boys with FXS. All of the participants were recruited from a related longitudinal study of boys with FXS or referred from physicians’ offices, genetic clinics, or developmental clinics in the eastern section of the United States.

The overall sample for this study included 45 boys with FXS for whom the diagnosis of full mutation FXS was confirmed by a DNA analysis. Enrollment criteria for the boys with FXS was an age of less than 16 years, a language level of at least 40 expressive words, and emergent word combinations (mean length of utterance greater than 1.1). The boys with FXS were further categorized as having Autism/no Autism as described below. We excluded boys whose primary language in the homes was not English, boys whose hearing threshold was greater than 25 dB HL in the better ear across 500, 1000, 2000, and 4000 Hz, and boys whose primary mode of communication was sign language. Approximately 75.4% of the boys with FXS were receiving some form of psychotropic medication, largely for attention-related problems.

*FXS without Autism (FXS-Only)*. Eighteen boys with FXS without Autism between the ages of 2.86 years and 13.88 years (*M* = 9.76, *SD* = 2.76) at the first assessment participated in the study. The mean Brief IQ composite of the *Leiter-R* fell within the mild range of intellectual disabilities (*M* = 59.1, *SD* = 16.7). Over seventy-two percent (72.2%) of the boys were Caucasian, 22.2% were African American, and 5.6% were other races/ethnicities. Maternal education ranged from 12 to 16 years (*M* = 13.7, *SD* = 1.6), with the terminal education level being a high school degree for 33.3% of mothers, and some college or a college degree for about two-thirds of the sample (66.7%).

*FXS with Autism (FXS-Autism)*. Twenty-seven boys with FXS and ASD between ages 3.5 years to 13.9 years (*M* = 8.9, *SD* = 2.9) at the first assessment participated in the study. The mean Brief IQ composite of the *Leiter-R* was within the moderate range of intellectual disabilities (*M* = 53.1, *SD* = 12.3). Approximately 85% (85.2%) of the boys were Caucasian, 11.1% were African American, and 3.7% were other races/ethnicities. Maternal education ranged from 12 to 20 years (*M* = 14.9, *SD* = 2.6), with the terminal education level being a high school degree for about a quarter of the mothers (25.9%) of this subsample, and some college or a college degree for about three-quarters (74.1%).

### 2.2. Measures

To address the primary research questions posited, this study employed three major outcome variables measuring speech, expressive vocabulary, and receptive vocabulary. The *Goldman-Fristoe Test of Articulation-2* (GFTA-2) was used to measure speech, while the vocabulary outcomes measures included the *Peabody Picture Vocabulary Test-III* (PPVT-III) and the *Expressive Vocabulary Test* (EVT). These measures were collected at three different time points, once at baseline and at two subsequent yearly intervals.

The *Goldman-Fristoe Test of Articulation-Second Edition* (GFTA-2) [29] was used to assess articulation at the single word level. The participant is shown a picture and is asked to produce a single-word response to label the picture. The GFTA-2 measures the ability of the individual to produce all of the English consonants in the initial, medial, and final positions in common words with an overall age-equivalent being generated.

Expressive vocabulary was assessed with the *Expressive Vocabulary Test (EVT*) [30]. The individual is asked to label a picture or give a synonym for the word provided by the examiner that also labels the picture. The EVT was standardized on the same sample as the *PPVT-III.* Levels of reliability are high (alpha coefficient = 0.95), and the *EVT* has adequate levels of validity correlating moderately to highly with other measures of language and intelligence. An age equivalent for each child’s performance was computed using published norms.

The *Peabody Picture Vocabulary Test-III (PPVT-III*) [31] was used to assess receptive vocabulary. Here, the individual is asked to select the visual representation of a word provided by the examiner from among four choices. The *PPVT-III* was standardized on 2725 individuals ranging in age from 2.5 to over 90 years. Levels of reliability are high (alpha coefficients = 0.95), and validity is strong as it correlates highly with other measures of language and intelligence. An age equivalent score was calculated using published norms.

### 2.3. Targeted Covariates

Four potential covariates were examined as possible contributors to the development of these speech and language outcomes. These included Leiter-R age equivalent, Autism status, maternal education, and level of FMRP. These variables were selected given their importance to the functioning of young boys with FXS. These variables also were considered time-invariant covariates given that all of these data were collected at the baseline assessment.

The *Leiter International Performance Scale-Revised (Leiter-R*) was used to assess nonverbal cognition [32]. The four subtests that comprise the Brief IQ Composite include Figure Ground, Form Completion, Sequential Order, and Repeated Patterns. The *Leiter-R* was standardized on 1719 individuals 2 to 20 years of age. Levels of reliability are high, with a test-retest coefficient of 0.96 for the Brief IQ Composite. The *Leiter-R* has adequate validity, correlating strongly (0.85 to 0.86) with other widely used IQ tests. The Brief IQ Composite was computed for each child using published norms, and the associated age equivalent was used in the data analyses.

The boys with FXS were classified as having Autism using the *Autism Diagnostic Observation Schedule-General (ADOS)* [33]. The *ADOS* is a standardized observation of children’s communicative and social behavior, and discriminates ASD from other developmental disorders as well as from normal behavior. The examiner interacts with the child for approximately 45 min in a series of structured and semi-structured activities in which the child is given opportunities to exhibit behaviors indicative of Autism. Trained examiners scored the tapes, and reliability computed on 20% of the boys was 0.89 for the individual items (range 0.83 to 0.96) and 0.93 on diagnosis (range 0.81 to 1.00).

Levels of FMRP were determined via DNA studies performed at Kimball Genetics in Denver, CO, using Southern blot and PCR analysis [27]. Blood smears were obtained from each individual and subsequently analyzed for FMRP employing the immunocytochemistry approach developed by Willemsen et al. [34]. For each participant, 200 lymphocytes were scored for the presence or absence of FMRP, thus providing an estimate of the percentage of lymphocytes expressing FMRP in the brain. For the sample, the average percentage of lymphocytes expressing FMRP indicated a low level of FMRP, with a range from 1% to 40% (M = 8.60%, SD = 8.03). FMRP levels were subjected to a log transformation for data analyses in order to adjust the distribution of the FMRP levels for the entire sample.

*Maternal education* was obtained by parent/guardian interview at entry into the study, and was determined by the number of years of education completed by the caregiver.

### 2.4. Procedures and Data Analyses

All data were collected at designated time points over the course of the study, with most children receiving their follow-up evaluations at the approximate anniversary date from their baseline assessment. All measures were administered by trained evaluators who were supervised by a speech-language pathologist and a child neuropsychologist.

To address the primary research question, three statistical procedures were conducted—one for each of the targeted outcomes. Given the repeated measures over time (time nested in child), we applied Hierarchical Linear Model (HLM), also known as mixed models, with developmental age as the index of time to assess linear growth in the three outcomes (GFTA-2 Age Equivalent, EVT Age Equivalent, PPVT-III Age Equivalent) over time. In addition, each procedure contained FMRP, Leiter-R Brief IQ age equivalent, FXS Group (FXS-Only, FXS-Autism), and maternal education as covariates. This permitted the examination of main effects as well as the possibility of two-way and three-way interactions. In the event of the presence of any two-way or three-way interaction, the continuous variables of Leiter-R age equivalents and log FMRP values were calculated by using a ±0.5 of a standard deviation for the Leiter-R (approximately 56.5 to 67.05) and for the log of FMRP (approximately 1.61 to 2.37) as a matter of both convenience and interpretability.

## 3. Results

Means and standard deviations are provided in Table 1 for the FXS-Only and the FXS-Autism groups at each of the three time points for chronological age, each of the covariates—including the log transformed FMRP, and the three outcome measures. Pearson correlations between the three outcome variables and the contributors were weak (*r* = 0.10 for maternal education and EVT) to moderate (*r* = 0.43 for FMRP and PPVT-III). Results are reported separately for the GFTA-2, EVT, and PPVT-III. For all outcomes, higher order interactions take precedence over any lower order interactions and main effects.

### 3.1. Speech Articulation

**Figure 1:**
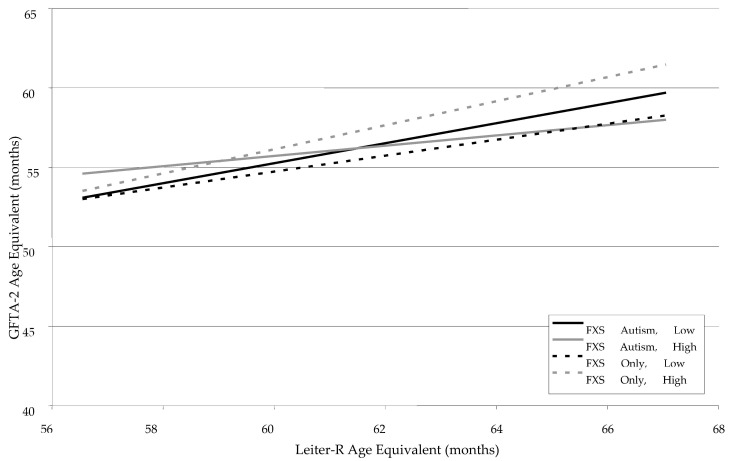
Developmental trajectories for single-word speech articulation (GFTA-2) as a function of Leiter-R Age Equivalent, FMRP Group, and Autism Status.

The GFTA-2 was used to measure single-word speech and articulation in the two groups. As can be seen in Table 1, the age equivalents for the GFTA-2 were not different between the two groups upon visual inspection. When each of the covariates was entered into the HLM, an interesting pattern of results emerged. As can be seen in Table 2, while there were main effects for Leiter-R age equivalent, indicating that GFTA-2 scores increased with advancing Leiter-R age equivalent, *F* (1, 89) = 24.19, *p* < 0.0001, Leiter-R effects were moderated in a three-way interaction between Leiter-R, FMRP level, and Autism status, *F* (1, 89) = 7.73, *p* < 0.007. Specifically, as can be seen in Figure 1, the pattern of results in the FXS-Only Group showed a somewhat flatter slope for lower levels of FMRP. In the FXS-Autism group, however, higher FMRP scores were associated with a reduced Leiter-R effect, suggesting that when ASD is present, higher FMRP levels are associated with a weaker correlation between Leiter-R mental age and GFTA-2 than lower levels of FMRP. When Autism is not present, this pattern is reversed such that the Leiter-R mental age slope on GFTA-2 is steeper as FMRP increases. In general, the relationship between Leiter-R age equivalent and GFTA-2 is positive across both groups; however, for the FXS-Only group, the slope of Leiter-R on GFTA-2 becomes less steep as FMRP decreases. Maternal education was not a factor in predicting GFTA-2 slope.

### 3.2. Expressive Vocabulary

**Figure 2:**
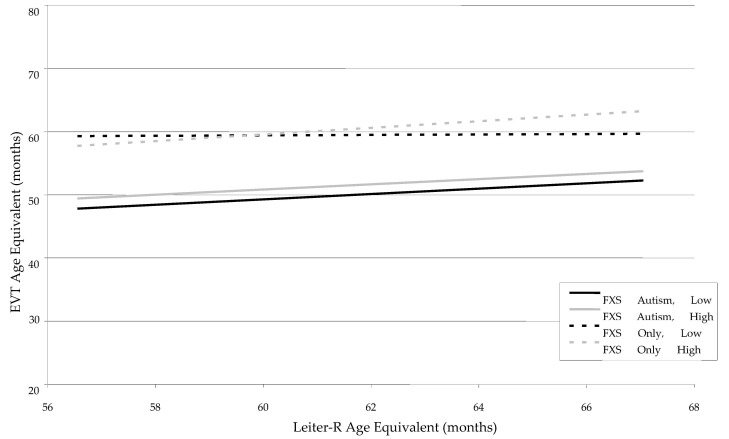
Developmental trajectories for expressive vocabulary (EVT) as a function of Leiter-R Age Equivalent, FMRP Group, and Autism Status.

The EVT was used to measure expressive vocabulary in the two groups. As can be seen in Table 1, the FXS-Autism Group scored significantly lower than the FXS-Only Group at each of the three time points upon visual inspection. When each of the covariates was entered into the HLM, main effects were noted for Autism status, *F* (1, 40) = 4.29, *p* < 0.04, and Leiter-R Age Equivalent, *F* (1, 96) = 8.28, *p* < 0.005, but these main effects were minimized by the presence of a two-way interaction between Leiter-R and FMRP, *F* (1, 96) = 6.27, *p* < 0.01, as well a three-way interaction between Leiter-R, FMRP, and Autism status, *F* (1, 96) = 7.01, *p* < 0.009 (see Table 3). Figure 2 indicates that the FXS-Only Group scored higher than the FXS-Autism Group on EVT, but this effect was moderated by FMRP and Leiter-R age equivalent. For all but the FXS-Only Group with low FMRP, increases in the Leiter-R age equivalents were associated with increases in the EVT age equivalent scores. At low levels of FMRP, however, the relationship is relatively flat, and the slopes are effectively parallel, indicating less of an impact of Leiter-R age equivalent on expressive vocabulary and a moderating effect of FMRP. Again, no impact of maternal education was present in the model.

### 3.3. Receptive Vocabulary

**Figure 3:**
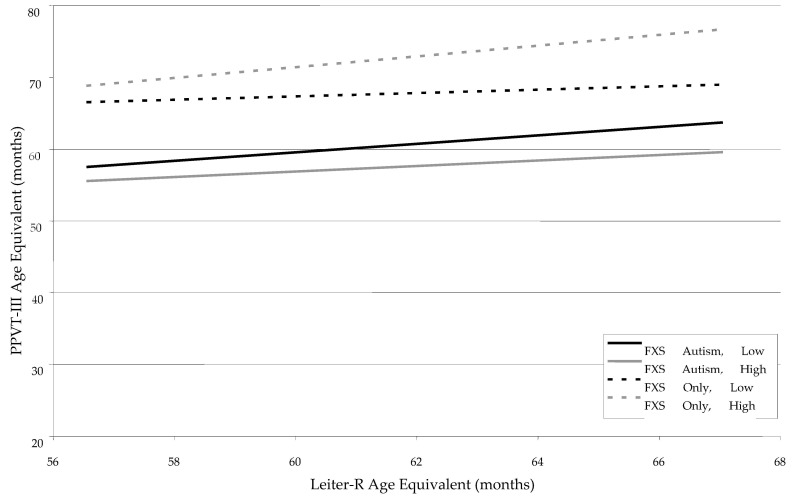
Developmental trajectories for receptive vocabulary (PPVT-III) as a function of Leiter-R Age Equivalent, FMRP Group, and Autism Status.

The PPVT-III was employed as the measure of receptive vocabulary for the two groups. A visual inspection of the mean scores across the groups in Table 1 suggests that the FXS-Only Group performed at a higher level than the FXS-Autism Group at each of the three time points. In Table 4, main effects were significant and positive for Leiter-R age equivalents, *F* (1, 91) = 9.43, *p* < 0.003, suggesting that as Leiter-R increased so did the PPVT-III scores. As with both the GFTA-2 and EVT, however, these main effects were minimized by the presence of a significant three-way interaction between Leiter-R, FMRP, and Autism status, *F* (1, 91) = 5.55, *p* < 0.02. As can be seen in Figure 3, while the lines for the FXS-Only Group do not cross, the low FMRP line is notably flatter than the high FMRP line. Again, this indicates that while there is a positive relationship between Leiter-R age equivalent and the PPVT-III, levels of FMRP serve as a moderator in weakening this relationship.

## 4. Discussion

This study examined speech and vocabulary growth in young boys with FXS, with a particular focus on how FMRP would contribute to this growth over time while controlling for several key factors (i.e., Autism status, nonverbal abilities, maternal education). It was hypothesized that FMRP would be positively related to the development of these speech, receptive vocabulary, and expressive vocabulary communication skills, with perhaps the interaction of FMRP with Autism status reflecting a differential pattern of influence. Findings revealed the presence of relatively rare occurring three-way interactions between the covariates for all three outcome variables. Specifically, the results suggested that FMRP did not show a direct relationship with the development of speech, expressive vocabulary, and receptive vocabulary; but, rather, the amount of FMRP moderated the relationship between mental age, Autism status, and each of the speech and vocabulary outcomes. In each instance, while there appeared to be a relationship between each of the outcome measures and mental age and, to some extent Autism status, these relationships were moderated by the level of FMRP. Specifically, higher levels of FMRP seemed to increase the relationship between developmental level and each of the speech and vocabulary outcomes for boys with FXS with and without Autism; i.e., no difference in trajectories between the groups; however, at lower levels of FMRP, these relationships seemed to weaken significantly for both groups.

In general, these findings were consistent with previous work [5,27] showing increased levels of FMRP to be associated with development and language-related functions; however, whereas we did not show a direct association between FMRP and speech and language functions, we did uncover three-way interactions for each of the outcomes suggesting the importance of FMRP in moderating the relationship between Autism status [19], developmental level, and speech and vocabulary development in boys with FXS. These findings highlight the complexities inherent in examining potential contributors to communication functions in young boys with FXS. These findings also suggest that it may be necessary for an individual to have a certain threshold level of FMRP prior to other variables (e.g., Autism status or mental age) having an impact on speech and language outcomes.

One surprising finding from this study was the relative lack of influence of maternal education on the three language-related outcomes. A number of studies have noted the importance of maternal education to language development e.g., [34], and the influence of the environment on behavior more generally, in individuals with FXS [35]; however, this relationship was not uncovered in this study of young boys with FXS with and without Autism. While Roberts et al. [21] did find a relationship of maternal education with receptive and expressive vocabulary, but not speech functions, the present study showed little relationship to the speech and vocabulary outcomes. It is unclear why this is the case, but could be related to the relatively higher maternal education level in the overall sample, with about 67% to 74% of each group having some college education or more, such that little variability was present between the groups. It may be the case that the use of other multidimensional measures of socioeconomic status and social determinants of health would produce different findings. We also submit that this lack of association could be due to our relatively small sample size.

Another interesting finding related to the main effects seen for Autism status. Previous findings have shown that the presence of Autism contributed to lower functioning on each of the outcomes [5,19,36]; however, this was not the case in the current study. There was a main effect seen for Autism status for both speech and receptive vocabulary, but not for expressive vocabulary. Further, the impact of Autism status was moderated by the level of FMRP and Leiter-R age equivalent. Again, suggesting the need for using a number of covariates in the exploration of speech and language-related functions in young boys with FXS.

Finally, it is important to note that the relatively small sample size ascertained for this study precludes large-scale generalization of these findings. While the pattern of findings was intriguing, especially the uncovering of the three-way interactions between developmental level, FMRP, and Autism status, a replication of these findings is in order with a larger sample size to further substantiate these relationships with speech and vocabulary development. In contrast, it could be argued that our findings actually reflect a more conservative set of results given that our exclusion criteria may have eliminated the most severely involved children (i.e., those with the lowest language abilities) which, in turn, may have increased the impact of FMRP on the speech and language trajectories. Additionally, we are aware that our three-way interactions may reflect an artifact of our sample size (i.e., Type 1 error) and associated power, but believe these findings provide a novel and compelling set of findings to support future work examining these relationships over time.

This study maintains a number of strengths, such as a well-described sample of full mutation young boys with FXS and a longitudinal design to examine relationships over time; however, there also were a number of study limitations. Specifically, as a retrospective study, we were limited in the nature of our measurement. First, while our measures of speech and language were reliable and valid at the time, there are now more current versions of these tools. While we do not anticipate that these newer versions would change the pattern of the observed relationships, as the overall format and content of the measures have remained the same, the magnitude of these relationships may be different. Second, our quantification of FMRP was current at the time, but there are now more sophisticated measurement strategies to obtain FMRP [2,3,28], likely producing more precise and reliable results and better reflecting brain expression in this protein; moreover, given the retrospective nature of this investigation, we do not have any opportunity to obtain newer samples from these participants. While the increased level of precision from contemporary measures of FMRP may contribute to a different pattern of findings, it also might show FMRP to be an even stronger moderating factor. Third, our study also did not include other variables that could have impacted the findings, such as the various types of therapies that these children may have been receiving (e.g., speech therapy, special education, pharmacological treatments) or many social determinants of health beyond maternal education. While we did not want to unduly stress our statistical model via overparameterizing given our small sample size, these factors remain important considerations for inclusion in examining the relationship between FMRP and speech and language development, and our findings hopefully lay the foundation for future work. Finally, although we did have a well-characterized sample of young boys with full mutation FXS, the sample was relatively small, thus warranting replication of these findings in future work.

## 5. Conclusions

In summary, this study examined the role of FMRP in the development of speech, expressive vocabulary, and receptive vocabulary in young boys with FXS when controlling for a number of key factors (i.e., nonverbal abilities, Autism status, maternal education). In general, while there appeared to be a relationship between each of the outcome measures and developmental level and, to some extent Autism status, these relationships were moderated by the level of FMRP. Specifically, higher levels of FMRP seemed to increase the relationship between developmental level and each of the outcomes for boys with FXS with and without Autism; however, at lower levels of FMRP, these relationships seemed to weaken significantly for both groups. These findings implicate increased complexity in examining the relationship between various contributors to the rates of growth of speech articulation, expressive vocabulary, and receptive vocabulary in young boys with FXS. In addition to replicating the current findings, future studies should address the whether FMRP levels affect speech and language differentially at different developmental epochs (e.g., perhaps studying more restricted age bands). While we did study how FMRP could affect the development of speech and language abilities, targeting its differential impact over time will require a novel study designed to directly address this question. Further, future research should examine how FMRP interacts with other constellations of variables in the manifestation of other aspects of speech and language (e.g., syntax development, phonological processes), cognitive abilities (e.g., executive functions, working memory), and related functional outcomes (e.g., adaptive behavior) in young boys with FXS, thus continuing to support potential intervention efforts for young boys with FXS [37,38].

## Figures and Tables

**Table 1:** Means and Standard Deviations for Speech Articulation, Expressive Vocabulary, Receptive Vocabulary, and Model Covariates for the FXS-Only and FXS-Autism Groups Across the Three Time Points.

Variable/Visit	FXS-Only		FXS-Autism	
	n	Mean (SD)	n	Mean (SD)
Chronological Age				
Visit 1	18	9.76 (2.76)	27	8.87 (2.93)
Visit 2	18	10.79 (2.77)	23	10.02 (3.17)
Visit 3	16	11.74 (2.82)	21	11.23 (3.10)
Maternal Education ^a^				
Visit 1	18	13.72 (1.56)	25	14.84 (2.66)
Visit 2	18	13.72 (1.56)	25	14.84 (2.59)
Visit 3	16	13.81 (1.60)	21	15.24 (2.70)
log FMRP ^a^				
Visit 1	18	2.08 (0.66)	25	1.85 (0.80)
Visit 2	18	2.08 (0.66)	25	1.85 (0.80)
Visit 3	16	2.07 (0.70)	21	1.95 (0.75)
Leiter-R Age Equivalent ^a^				
Visit 1	17	61.94 (11.25)	24	52.92 (14.18)
Visit 2	15	67.07 (5.86)	17	59.88 (10.03)
Visit 3	16	66.63 (7.22)	21	60.19 (8.77)
GFTA-2 Age Equivalent				
Visit 1	17	53.76 (12.51)	22	53.77 (15.43)
Visit 2	18	58.06 (15.63)	23	54.13 (13.85)
Visit 3	15	59.13 (13.45)	20	59.80 (14.88)
EVT Age Equivalent				
Visit 1	17	56.94 (18.48)	23	47.48 (15.39)
Visit 2	18	59.39 (17.10)	25	47.64 (15.93)
Visit 3	16	63.69 (17.60)	21	53.43 (13.88)
PPVT-III Age Equivalent				
Visit 1	15	73.80 (15.83)	22	57.18 (23.30)
Visit 2	18	73.50 (24.40)	23	56.48 (25.49)
Visit 3	15	77.60 (19.58)	21	62.71 (25.69)

Note. ^a^ log FMRP, Leiter-R Brief IQ Age Equivalent, and Maternal Education in years of school completed were all collected at the time of enrollment; GRTA-2 = *Goldman-Fristoe Test of Articulation-Second* Edition; PPVT = *Peabody Picture Vocabulary Test-III*; EVT = *Expressive Vocabulary Test*.

**Table 2:** The impact of FMRP on the GFTA-2 age equivalent over time when controlling for maternal education, Autism status, and Leiter-R IQ age equivalent.

Effect	Estimate	Std Error	DF	F Value	*p*-Value
Intercept	56.57	2.98	1, 39	882.27	<0.0001
Mother’s Education	1.04	0.83	1, 39	1.54	0.22
Autism Status *	−0.22	3.93	1, 39	0.00	0.96
Leiter-R IQ Age Equivalent	0.63	0.18	1, 89	24.19	<0.0001
Log transformed FMRP	2.45	4.57	1, 39	0.18	0.68
Leiter-R X Autism Status *	−0.15	0.23	1, 89	0.46	0.50
Log transformed FMRP X Autism Status *	−2.57	5.63	1, 39	0.21	0.65
Leiter-R X FMRP	0.34	0.20	1, 89	0.06	0.81
Leiter-R X FMRP X Autism Status *	−0.74	0.57	1, 89	7.73	0.007

* FXS-Autism is the reference group.

**Table 3:** The impact of FMRP on the EVT age equivalent over time when controlling for maternal education, Autism status, and Leiter-R IQ age equivalent.

Effect	Estimate	Std Error	DF	F Value	*p*-Value
Intercept	60.10	3.42	1, 40	653.19	<0.0001
Mother’s Education	1.42	0.95	1, 40	2.25	0.14
Autism Status *	−9.27	4.48	1, 40	4.29	0.04
Leiter-R IQ Age Equivalent	0.26	0.21	1, 96	8.28	0.005
Log transformed FMRP	1.14	5.23	1, 40	0.25	0.62
Leiter-R X Autism Status *	0.15	0.24	1, 96	0.43	0.52
Log transformed FMRP X Autism Status *	0.89	6.36	1, 40	0.02	0.89
Leiter-R X FMRP	0.69	0.21	1, 96	6.27	0.01
Leiter-R X FMRP X Autism Status *	−0.71	0.27	1, 96	7.01	0.009

* FXS-Autism is the reference group.

**Table 4:** The impact of FMRP on the PPVT-III age equivalent over time when controlling for maternal education, Autism status, and Leiter-R IQ age equivalent.

Effect	B Estimate	Std Error	DF	F Value	*p*-Value
Intercept	70.28	5.18	1, 39	387.13	<0.0001
Mother’s Education	0.14	1.44	1, 39	0.01	0.92
Autism Status *	−11.19	6.80	1, 39	2.70	0.11
Leiter-R IQ Age Equivalent	0.49	0.27	1, 91	9.43	0.003
Log transformed FMRP	6.59	8.18	1, 39	0.07	0.79
Leiter-R X Autism Status *	0.00	0.32	1, 91	0.00	0.99
Log transformed FMRP X Autism Status *	−10.61	9.90	1, 39	1.15	0.29
Leiter-R X FMRP	0.67	0.32	1, 91	0.98	0.32
Leiter-R X FMRP X Autism Status *	−0.95	0.40	1, 91	5.55	0.02

* FXS-Autism is the reference group.

## Data Availability

The data presented in this study are housed at Frank Porter Graham Child Development Institute and are available from the first author upon request.
